# RCT of an integrated CBT-HIV intervention on depressive symptoms and HIV risk

**DOI:** 10.1371/journal.pone.0187180

**Published:** 2017-12-14

**Authors:** Karin Tobin, Melissa A. Davey-Rothwell, Bareng A. S. Nonyane, Amy Knowlton, Lawrence Wissow, Carl A. Latkin

**Affiliations:** 1 Department of Health, Behavior and Society, Bloomberg School of Public Health, Johns Hopkins University, Baltimore, MD, United States; 2 Department of Biostatistics, Bloomberg School of Public Health, Johns Hopkins University, Baltimore, MD, United States; University of New South Wales, AUSTRALIA

## Abstract

Depression and depressive symptoms mediate the association between drug use and HIV risk. Yet, there are few interventions that target depressive symptoms and HIV risk for people who use drugs (PWUD). This study was a randomized controlled trial of an integrated cognitive behavioral therapy and HIV prevention intervention to reduce depressive symptoms, injection risk behaviors and increase condom use in a sample of urban people who used heroin or cocaine in the prior 6 months. A total of 315 individuals aged 18–55, who self-reported at least one HIV drug and sex risk behavior and scored ≥16 and <40 on the Centers for Epidemiologic Studies-Depression (CES-D) scale were randomized using a two-block design, stratified by sex to ensure equivalent numbers, to a 10 session intervention arm (n = 162) or a single session control arm (n = 153). The outcomes of interest were decreases in CES-D score and injection risk behaviors and increases in condom use. The sample was majority African American (85%) and unemployed (94%). Nearly half (47%) reported injection in the prior 6 months and only 19% were taking medication for depression. Follow-up assessments were conducted at 6 and 12 months. Retention at 12 months was 94%. Intervention arm was associated with statistically significantly lower CES-D score at 12 month compared to control. No differences were observed between arms in injection risk. At 6 month, intervention was associated with greater odds of condom use with non-main partner. These findings suggest the potential role of the integrated intervention in reducing depressive symptoms, but weak impact on HIV risk. This trial is registered with ClinicalTrials.gov under the title “Neighborhoods, Networks, Depression, and HIV Risk” number NCT01380613.

## Introduction

People who use illicit substances, such as heroin and cocaine, bear a significant burden of HIV [1] and injection drug use remains a major risk factor for HIV transmission in the United States [2]. Studies have shown that depression and depressive symptoms mediate the association between drug use and HIV risk [3,4]. There remains a lack of integrated interventions that target depressive symptoms and HIV risk behaviors. Addressing both simultaneously may serve as primary and secondary HIV prevention strategy that can impact people who use drugs and their risk partners, as well as improving mental health outcomes.

Cognitive behavioral therapy (CBT) is well established as an efficacious approach for reducing depressive symptoms [5]. CBT emphasizes the modification of maladaptive perceptions, behaviors and emotions that lead to and reinforce depressive symptoms. A number of trials have been conducted to test the efficacy of integrated CBT-substance use interventions and have demonstrated improvements in depression, coping and reduced substance use [6–8]. Yet, many of these studies only assessed effects at 6 month follow-up and there is a need to determine long-term sustainability of effects.

In this manuscript we describe a randomized controlled trial of an integrated intervention (herein referred to as Workshop) delivered by lay facilitators that aimed to train individuals who use drugs and experience depressive symptoms in cognitive behavioral therapy skills and injection and sexual risk reduction strategies. We hypothesized that compared to a control arm, Workshop would result in reduced depressive symptoms, reduced injection risk behaviors and reduced sexual risk (e.g. increased condom use) at 6 and 12 month follow-up.

## Materials and methods

### Study design

This was a two condition randomized controlled trial conducted in Baltimore, Maryland, ranked amongst the top cities for HIV prevalence and unrecognized infection compared to 20 other metropolitan areas [9,10]. Recruitment and enrollment occurred from March, 2010 to January, 2012 with assessments at baseline, 6 and 12 months. All study activities were conducted at a research clinic located in a mixed residential and commercial neighborhood. This trial is registered with ClinicalTrials.gov under the title “Neighborhoods, Networks, Depression, and HIV Risk” number NCT01380613. Due to administrative delays the posted date of the application to ClinicalTrials.gov is March, 2011.

### Sample and procedures

Recruitment was conducted using a variety of methods, including street-based outreach, word-of-mouth, flyers, advertisements in local papers and referrals from community agencies. Participants were screened for eligibility by research staff via telephone or in-person at the research clinic. Inclusion criteria for enrollment was: 1) aged 18–55; 2) willingness to attend group sessions; and 3) at least one drug related HIV risk behavior defined as a) self-report injection drug use 3 or more times in the past week, or b) crack use in the prior 6 months; and 4) at least one sexual risk behavior, defined as a) 2 or more sex partners in the past 90 days or b) having a sex partner who injected drugs or smoked crack or c) sex partner is HIV positive. Only participants who scored ≥16 and <40 on the Center for Epidemiologic Studies–Depression scale (CES-D), were randomized [11].

**Fig 1:**
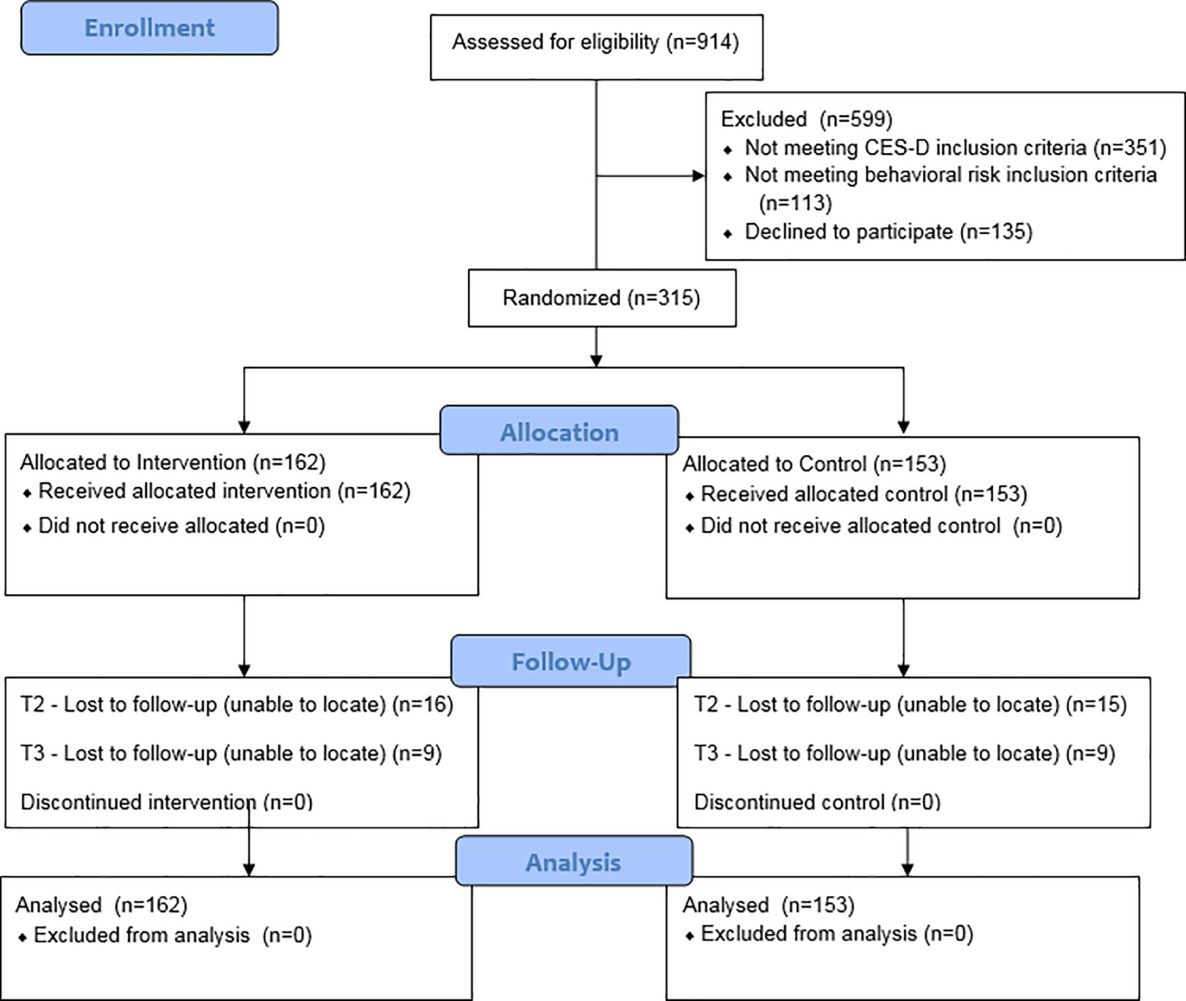
CONSORT flow diagram of the Workshop study.

Participants were excluded if they reported being enrolled in another HIV prevention or depression study in the past 3 years, if they scored 40 or over on the CES-D or reported acute psychiatric symptoms such as active psychosis or suicidal ideation (Fig 1). Exclusion based on CES-D score >40 was based on recommendations of a psychiatrist who was consulting on the study. Participants who were not randomized (n = 135) were retained in the prospective component of the study but were not included in the present analysis.

Baseline procedures consisted of written informed consent, a HIV and substance use risk assessment using audio-computer self-administered programming (ACASI) and an interviewer administered survey to assess depressive symptoms. Participants provided documentation of their HIV seropositive status or were tested for HIV antibodies using rapid Orasure^TM^ specimen collection device. All participants tested received pre and post-testing risk reduction counseling by trained staff.

### Randomization

Participants eligible for randomization were informed of the date that randomization would occur. Reminder phone calls and letters were sent to participants. The study Data Manager used a computerized program to assign individuals, in the order that they arrived, to the experimental or control arm using a two-block design, stratified by sex to ensure equivalent numbers in the two arms.

### Follow-up data collection

Follow-up periods were at 6 and 12 months after the last session of the intervention or control condition (T2 and T3, respectively). Retention strategies included phone calls and letters to the participant and their contacts, street-based outreach to residences and public databases to identify participants who were incarcerated. The follow-up visits used the same surveys and methods as the baseline visit. Interviewers were blinded to the study condition. Participants received $35 for each follow-up visit.

### The Workshop (intervention arm)

The Workshop was a 10 session (9 group-based format and 1 individual-format) that was delivered over five weeks in 90 minute sessions. Group sizes ranged from 4–10 individuals and were mixed gender. Workshop was informed by a pilot CBT intervention that was found to be acceptable by people who inject drugs (PWID) in Baltimore [12] and two efficacious peer-influence HIV prevention interventions [13,14]. Consistent with the principals of CBT, the Workshop aimed to train individuals in techniques to: a) identify negative cognitions and social and environmental stressors, b) restructure cognitions, social interactions and environments to improve mood, c) plan non-drug/alcohol based activities that improved mood, and d) reduce sexual and drug-related risk behaviors. (See S1 Table for an outline of session content). HIV risk reduction skills included practice of condom use and skills for reducing HIV and hepatitis C risk associated with injection drug use and sharing drugs (e.g. crack pipes). Attendance of intervention sessions was good, with the majority (76%) attending 80% of the sessions. Participants received $25 for each session they attended. All sessions were audio-recorded for supervision and fidelity monitoring.

#### Facilitator training and fidelity

Sessions were conducted by male and female co-facilitators who had 12 years of education (HS diploma or GED equivalent) and an average of 10 years of experience leading group-based HIV prevention interventions with people who use drugs. They were trained by the lead author which entailed didactic educational sessions on depressive symptoms, CBT and HIV risk reduction; review and discussions about the curriculum; and observed practice implementing the curriculum.

After each session, facilitators completed session summary forms that were reviewed by the lead author. Weekly supervision meetings were also held to discuss the sessions and problems encountered. To monitor fidelity a random selection of 10% of the audio-recorded sessions were reviewed by the lead author and evaluated based on adherence to curriculum scripting and activities.

### Control arm

The control arm consisted of a one-hour group session in which a trained female facilitator presented various mental health resources available in the community. Participants received $25 for completing the group session.

### Measures

#### Center for epidemiologic studies–depression scale (CES-D)

As the focus of the intervention was on reducing depressive symptoms, changes to CES-D score was the outcome of interest. Depressive symptoms were assessed using the CES-D; a 20-item, 4-point scale developed for use in the general population [15]. The scale has high validity and reliability[16]. The CES-D has been shown to have a high sensitivity for Diagnostic Statistical Manual version IV (DSM-IV) major depression and an adequate specificity as a screening instrument for depression [17]. A cut-off score of 16 or greater has been validated as an indication of probable clinical depression in the general population [18]. The Cronbach's alpha was 0.92 at baseline.

#### Substance use and injection-risk behaviors

History of illicit drug use and injection drug use in the prior 6 months were collected by trained interviewers. Injection-risk behaviors were assessed using ACASI. Injection-risk was determined based on questions about *needle sharing* (using a needle after at least one person or using a needle that was not clean), *injection equipment sharing* (using a cooker or rinse water after someone else had used it) and drug *splitting* behaviors (using an unclean syringe to measure out drugs or using drugs that had been squirted into a cooker from another persons’ syringe). If a participant indicated any of the above behaviors, they were categorized as having an injection-risk behavior (coded as 1) versus no injection risk behavior (coded as 0).

#### Condom use

Workshop aimed to increase condom use to 100% as this is the most effective prevention strategy for reducing HIV sexual risk. Condom use during vaginal or anal sex with both main and non-main partners was assessed for the prior 90-day period using ACASI. Literature has indicated that condom use varies by partner type, such that use is lower in main partner relationships. Post-hoc, a binary sex risk variable was constructed separately for main and non-main partners: 0 indicated less than 100% condom use for vaginal or anal sex and 1 indicated 100% condom use or not having this type of sex.

#### Socio-demographics

Age, race, highest educational attainment, relationship status, homelessness in the prior 6 months and current employment status.

#### Mental health service utilization

Participants also reported whether they had ever been told by a doctor or mental health professional that they had depression (yes versus no); whether they had seen a mental health provider in the prior 6 months (yes versus no), and if they are currently taking medication for depression (yes versus no).

#### Statistical analyses

All randomized study participants (n = 315) were included in the analysis and were analyzed based upon arm assignment (i.e. intent to treat). T-test and chi-square statistics were used for continuous and categorical variables respectively to compare the arms based on demographic, mental health service utilization and drug use variables. There was only missing data on the baseline variable “told by a mental health provider that you had depression” and this variable was not included in the final multivariate model. Generalized estimating equations (GEE) models were fitted for the CES-D score, injection-risk behaviors and 100% condom use with main and non-main partner outcomes. These modeled the mean outcome over the three time points and the correlation between measurements within each participant. All models included randomization arm, time point, arm-by-time-point interaction and controlled for baseline homelessness and having seen a mental health provider. An unstructured correlation structure was assumed with robust variance estimators. The model of condom use with main partner was conducted with a sub-sample of participants who reported having a main partner (n = 260).

All protocols were approved by the Johns Hopkins Bloomberg School of Public Health Institutional Review Board on March 2009. This research was funded through a grant from the National Institutes of Drug Abuse grant 1R01 DA022961. This trial is registered with ClinicalTrials.gov under the title “Neighborhoods, Networks, Depression, and HIV Risk” number NCT01380613. The authors confirm that all ongoing and related trials for this intervention are registered.

## Results

Fig 1 depicts the CONSORT Flow Diagram of the trial. A total of 914 individuals were screened for eligibility. We did not record the total number of individuals who viewed our recruitment materials or were approached by our recruiter. Of the 914, 599 (66%) individuals were excluded: 351 did not meet the CES-D score ≥16 criteria, 113 did not meet the behavioral risk criteria and 135 declined to participate in the randomization process. Of the 315 who were randomized, 100% attended the first session. Attendance rates for the remaining nine sessions of the intervention condition were high: Session 2 (89%); 3 (86%); 4 (83%); 5 (84%); 6 (81%); 7 (82%); 8 (92%); 9 (84%); 10 (85%).

### Sample characteristics and randomization balance

Table 1 presents the demographic and risk behavior characteristics of *N* = 315 randomized participants. The majority were Black (85%), nearly two-thirds of the sample were male (57%), most had an educational attainment of 12 years/GED or more (59%), and half were single (53%). At baseline, a greater proportion of participants randomized to the intervention arm reported homelessness in the prior 6 months compared to control (45% versus 31%, respectively *p* < 0.01). Eleven percent of the sample self-reported HIV seropositive status.

**Table 1:** Baseline characteristics of randomized participants (n = 315) in the Workshop study, Baltimore Maryland.

	Total Sample	Intervention	Control	
	N = 315	N = 162	N = 153	
Variable	N (%)	N (%)	N (%)	p-value
**Mean CES-D score (SD)**	25.6 (6.42)	25.8 (6.45)	25.3 (6.40)	0.52
**Sex**				
Male	180 (57)	97 (60)	83 (54)	
Female	135 (43)	65 (40)	70 (46)	0.31
**Race**				
Black	269 (85)	137 (85)	132 (86)	
White	42 (13)	23 (23)	19 (19)	
Other	4 (1)	2 (1)	2 (2)	.90
**Mean age (SD)**	43.6 (7.30)	43.4 (7.23)	43.7 (7.41)	0.72
**Highest Education**				
1–11 years	129 (41)	68 (42)	61 (40)	
12^th^/HS/GED	147 (47)	77 (48)	70 (46)	
> = some college	39 (12)	17 (10)	22 (14)	0.58
**Relationship status**				
Married/committed	94 (30)	49 (30)	45 (29)	
Widowed/divorced/separated	53 (17)	28 (17)	25 (16)	
Single	168 (53)	85 (52)	83 (54)	0.95
**Homeless** (past 6 months)				
No	195 (62)	89 (55)	106 (69)	
Yes	120 (38)	73 (45)	47 (31)	0.01
**Current employment status**				
Employed (full or part-time)	20 (6)	8 (5)	12 (8)	
Unemployed (seeking work)	151 (48)	75 (46)	76 (50)	
Unemployed (not seeking work)	144 (46)	79 (49)	65 (42)	0.38
**Told by doctor/mental health professional had depression** (n = 174)				
No	89 (51)	48 (54)	41 (48)	
Yes	85 (49)	41 (46)	44 (52)	0.45
**Seen a mental health professional** (past 6 months)				
No	162 (51)	73 (45)	89 (58)	
Yes	153 (49)	89 (55)	64 (42)	0.02
**Taking medication for depression**				
No	256 (81)	134 (82)	122 (80)	
Yes	59 (19)	28 (17)	31 (20)	0.50
**HIV status**				
Negative	279 (89)	143 (89)	136 (89)	
Positive	34 (11)	17 (11)	17 (11)	0.89
**Use heroin or cocaine past 6 months** (yes)	315 (100)	162 (100)	153 (100)	1.00
**Injection drug use** (past 6 months)				
No	167 (53)	89 (55)	78 (51)	
Yes	147 (47)	72 (45)	75 (49)	0.45
**Use crack** (past 6 months)				
No	75 (24)	36 (22)	39 (25)	
Yes	240 (76)	126 (78)	114 (75)	0.50

The majority reported smoking crack in the past 6 months (76%), nearly half reported injection drug use (47%) and one-third reported both injection drug and crack use (*n* = 103; 32%). When examining baseline injection and use of crack, the majority of the sample reporting use at least weekly (88% injection) and (83% crack). Therefore we chose to dichotomize these variables. Among those who reported injecting at baseline, 90% reported injection-risk behaviors.

Approximately half of the sample (55%) had ever been told that they had a mental illness and nearly half (49%) of these indicated that they had been told the mental illness was depression. Half of the sample reported seeing a mental health professional in the prior 6 months (49%) and a greater proportion were randomized into the experimental arm (*p* = 0.02). Of note, this difference was not observed at the 6 or 12 month follow-up period. A minority (19%) of the entire sample reported currently taking medications to treat depression. There was no difference between study conditions on history of depression diagnosis (*p* = 0.45).

#### Effect of the Workshop on CES-D scores over time

Table 2 depicts changes in mean CES-D score over time by study arm and shows that both conditions had declines in symptom scores. Table 3 presents results from fitting the GEE models There was a significant reduction in CES-D score of the participants in the intervention arm at T2 and T3. Their mean CES-D score was reduced by 5 points (coefficient = -5.04, CI [-6.68,-3.39]) at T2, and by 6 units (coefficient = -5.90, CI [-7.75,-4.05]) at T3 compared to the baseline average. For the Workshop participants, the additional reduction in CES-D scores at T2 was not significantly greater than the reduction observed in the control group (coefficient = -1.13, CI [-3.49,-1.22]) whereas at T3 the additional reduction was statistically significantly greater than that of the control arm (coefficient -2.83, CI [-5.28,-0.38]).

**Table 2:** Changes in outcomes by arm at baseline, 6 and 12 month follow-up, the Workshop study, Baltimore Maryland.

	Baseline			T2			T3		
	Intervention	Control	p-value	Intervention	Control	p-value	Intervention	Control	p-value
**Mean CES-D** (SD)	25.8 (6.45)	25.3 (6.40)	0.52	19.7 (10.6)	20.2 (10.6)	0.67	17.1 (10.4)	19.6 (11.6)	0.06
**Injection risk over the past 6 months**									
Yes (n,%)	67 (42)	67 (44)	0.70	24 (17)	30 (22)	0.25	16 (10)	22 (15)	0.21
**Sex risk: 100% condom use with main partner over the last 90 days**									
Yes (n,%)	54 (33)	44 (29)	0.44	74 (51)	53 (39)	0.04	69 (45)	66 (46)	0.86
**Sex risk: 100% condom use with non-main partner over the last 90 days**									
Yes (n,%)	82 (51)	83 (55)	0.48	123 (85)	105 (77)	0.09	130 (86)	125 (87)	0.82

**Table 3:** GEE model results of intervention arm and time effect (n = 315) of Workshop intervention, Baltimore Maryland (n = 315).

			Time effect		Time*Arm interaction	
	Intercept	Arm effect	T2 vs. T1	T3 vs. T1	T2	T3
**Mean CES-D score**	25.3 [24.3, 26.3]	0.46 [-0.95, 1.88]	-5.04** [-6.68, -3.39]	-5.90** [-7.75, -4.05]	-1.13 [-3.49, 1.22]	-2.83* [-5.28, -0.38]
**Injection risk behaviors** (1 = yes risk)						
(Odds Ratios [95% confidence intervals])	0.78 [0.57, 1.07]	0.91 [0.58, 1.43]	**0.39** **[0.27, 0.56]**	**0.24** **[0.15, 0.36]**	0.75 [0.44, 1.27]	0.69 [0.35, 1.36]
**Sex risk main partner** (1 = yes risk)						
(Odds Ratios [95% confidence intervals])	0.14 [0.08, 0.24]	**1.88+** **[0.95, 3.74]**	3.60** [2.03, 6.37]	4.32** [2.47, 7.57]	0.96 [0.45, 2.08]	0.64 [0.31, 1.34]
**Sex risk non-main partner** (1 = yes risk)						
(Odds Ratios [95% confidence intervals])	1.24 [0.90, 1.71]	0.83 [0.53, 1.30]	2.65** [1.72, 4.08]	5.32** [3.15, 8.98]	1.99* [1.03, 3.83]	1.12 [0.54, 2.38]

CES-D effects are changes in CES-D score per unit change in the predictor, and injection and sex risk effects are expressed as odds ratios all models control for baseline having seen a mental health provider [+p-value< 0.10; *p-value<0.05; **p-value<0.001]

#### Effect of the Workshop on injection-risk behaviors

Table 2 presents the changes in proportion by arm reporting injection risk and Table 3 presents results from the GEE model and indicates that the overall model without the time-interaction term did not show a significant effect for the Workshop on injection risk behaviors (OR = 0.91, CI [0.58, 1.43]). There was a statistically significant time effect which is also depicted in Table 3 showing that both Workshop and control arms had reduced injection-risk behaviors at T2 and T3 compared to baseline.

#### Effect of the Workshop on condom use with main sex partner

The model of condom use with main partner was conducted with a subsample of participants who reported having a main partner (*n* = 260, 83% of the total sample). Table 3 presents results from the GEE model and indicates that the overall model without the time-interaction term showed a marginally significant effect for the Workshop on 100% condom use with main sex partner (OR = 1.88, CI[0.95, 3.74]). There was also a statistically significant time effect on 100% condom use with main partner for both Workshop and control arms at T2 and T3.

#### Effect of the Workshop on condom use with non-main sex partner

Results from the GEE model indicates that the overall model without the time-interaction term did not show a significant effect for the Workshop on 100% condom use with non-main sex partner (Table 3: OR = 0.83, CI [0.53–1.30]). There was a statistically significant time effect such that both Workshop and control arms had increased 100% condom use at T2 and T3. We also observed a significant time by arm effect at T2 indicating higher odds of 100% condom use with non-main partner among intervention arm compared to control arm (OR = 1.99, CI[1.03, 3.83]).

## Discussion

The goal of this RCT was to test the efficacy of the Workshop on reducing depressive symptoms and multiple HIV risk behaviors in a sample of predominately black individuals who use drugs and experience high depressive symptoms. We report that the Workshop had a statistically significant impact on reducing depressive symptoms compared to the control arm at 12 month follow-up. The Workshop utilized vivid metaphors and visual aids which may have enhanced processing and recall of the intervention materials and hence had a sustained impact on depressive symptoms. These results are consistent with other CBT-based interventions that have been tailored to the abilities and needs of impoverished and marginalized populations [19].

We observed a time-effect such that both groups had statistically lower CES-D scores at the follow-up periods compared to baseline. However, at T3 the Workshop continued to have reduced depressive symptoms. This “sleeper effect” has been observed in other studies and suggests the sustainability of CBT skills [5]. As a significant portion of the Workshop focused on skills for increasing social interactions with supportive individuals, the effects may have required more time for effects to be observed. The lack of difference between arms at T2 may be due to low statistical power. Alternatively the effect of the control condition, which reviewed mental health resources, may have prompted individuals to seek out support or mental health services. As the study staff, including the interviewers, were highly trained and had excellent rapport with participants, the study visits themselves may have had a therapeutic impact.

Another aim of the Workshop was to reduce injection-related risk behaviors but no differences between the arms were observed. One possible explanation for this may also be low power to detect small differences as only half of the sample reported injection at baseline. It is also likely that there was an insufficient dose of Workshop content focused on injection risk to have an effect compared to the control condition (who also received individual counseling at the baseline commensurate with standard HIV pre and post-test counseling). Future interventions should better integrate or increase dose of risk reduction into sessions.

The third focus of the Workshop was to reduce sexual risk behaviors. The Workshop had marginal effects on 100% condom use with main partners compared to the control but we did not observe differences in the time*arm interaction. There may have been insufficient dose of Workshop content focused on increasing condom use specifically with main partners. Changing sexual risk behaviors in main partnerships is hampered by issues of trust and power dynamics compared to other sexual contacts and effective intervention models tend to involve both partners to achieve behavior change. As there is inconsistent evidence on whether depression is associated with sexual risk behavior [20], it is also possible that decreasing depressive symptoms would not have an effect on condom use as much as individual risk reduction counseling, which both conditions received. However, it is notable that at 12 months, over half of the study participants continued to report unprotected vaginal and/or anal sex with their main partners suggesting the need to continue to focus on these dyadic risk behaviors. With regard to non-main sexual partners, we observed a statistically significant effect at T2 however this was not sustained at the 12 month period. We have observed similar results in condom use with non-main partners in a prior HIV prevention intervention[13]. It is also possible that sexual partners are a significant source of social support and as the Workshop focused on bolstering social support, participants may have spent more time with their partners and hence had the opportunity for unprotected sex.

There are a number of limitations of this study that should be noted. First, the lack of equal attention control diminishes our ability to conclude that behavioral changes were specific to the intervention content versus attention alone. We observed a statistically significant time effect for all outcomes which suggests that holding a single session that provides resources for addressing depression may be sufficient for short term improvements in CES-D and HIV risk behaviors. However, as a greater proportion of the participants in the intervention arm were homeless and had seen a mental health professional in the prior 6 months, this suggests that 10 sessions focused on skills building and practice may be a minimum amount to achieve a sustained decrease in depressive symptoms in marginalized populations. The difference in number of sessions also resulted in an imbalance of net monetary incentives. Notably, retention did not vary by arm which suggests that the incentives contributed to the excellent group attendance rates but may not have bolstered effects of the intervention.

Second, this was a convenience sample of older, predominately black people who use drugs, which included both injection and non-injection users. Therefore, our results may not be generalizable to people who use drugs in different geographic locations or younger populations. Third, this study relied on self-reported measures of injection drug and sexual risk behaviors. While we utilized ACASI to minimize bias, risk behaviors at follow-up may have been under-reported. Fourth, the two conditions were not balanced at baseline in terms of homelessness and seeing a mental health professional, which may have attenuated the Workshop effects. Finally, our study cannot determine whether or not drug use contributed to depression or whether depression preceded the drug use [21].

These limitations notwithstanding, the results of this trial suggest that the Workshop is an approach to reducing depressive symptoms and may affect injection drug use and sexual risk behaviors. Participants in this study were trained to use CBT skills without the psycho-educational methods of written self-assessments and workbook activities[22] and therefore is more accessible to populations with low literacy. The intervention was implemented by facilitators who were not mental health professionals. This may enable wider dissemination and implementation to community-based agencies and substance abuse treatment centers who cannot afford psychologists or licensed social workers. This study adds to other evidence-based programs that aim to target depression co-occurring with other health outcomes such as substance use[7,8,23] and improving medical adherence[24]. Such integrated approaches acknowledge the co-morbidities among substance users that ought to be addressed simultaneously given their often mutually reinforcing nature.

## Supporting information

S1 Table Description of Workshop sessions. (DOCX)

S1 File CONSORT checklist. (DOCX)

S2 File Workshop protocol. (DOCX)
