# Supplementary material for: RCT of an integrated CBT-HIV intervention on depressive symptoms and HIV risk
Source: PLoS One. 2017 Dec 14;12(12):e0187180. doi: 10.1371/journal.pone.0187180 (PMC5730221; doi:10.1371/journal.pone.0187180)
Supplement: S1 Table — (DOCX) [file pone.0187180.s001.docx]

Supplemental Table 1: Workshop Sessions

| Session | Format | Length | Description of session activities [Workshop components] | Personal Coach Assignment |
| --- | --- | --- | --- | --- |
| 1 | Group | 90 minutes | - Establish rapport. - Introduce the purpose of the Workshop: to train individuals to use CBT skills to help themselves feel better [Be a Personal Coach] | Remember a pleasant experience. |
| 2 | Group | 90 minutes | - Identifying and restructuring negative thoughts and social interactions [using Yellow and Blue metaphor*]. - [Using STOP mnemonic: Slow down, Thoughts (Yellow or Blue), Options, Practice] to practicing behavioral activation of cognitive restructuring skills | Write down two things you do to slow down |
| 3 | Group | 90 minutes | - Developing Personal Coach statements [as a way to restructure cognitions and give self credit for using the skills] - Practice STOP in different scenarios. | See how many situations you are able to use STOP. |
| 4 | Group | 90 minutes | - Identifying pleasant and supportive social network members [Yellow People]. - Setting a goal for spending time with a Yellow Person. | Choose someone from your Yellow people list and given them credit for the impact they have had on you. |
| 5 | Group | 90 minutes | - Identifying Yellow and Blue places. - Developing strategies for restructuring cognitions, feelings and behaviors in Blue places. | Spend time in a Yellow place. |
| 6 | Group | 90 minutes | - Review of sexual risk behaviors and prevention options. - Using STOP in sexually risk situations. | Share sex risk reduction options with someone. |
| 7 | Group | 90 minutes | - Review of HIV and HCV drug risk behaviors and prevention options (injection, snorting and smoking behaviors). - Using STOP in drug risk situations. | Share drug risk reduction options with someone. |
| 8 | Individual | 60-90 minutes | - Check-in about learning and using skills - Goals for using skills - Risk reduction goals |  |
| 9 | Group | 90 minutes | - Sustaining skills: STOP, Personal Coach statements, Yellow and Blue | Identify three options for sustaining the skills. |
| 10 | Group | 90 minutes | - Graduation ceremony giving credit for hard work and program completion. |  |

* *Yellow and Blue* was a metaphor used to personify and symbolically represent negative and maladaptive (Blue) thinking, people, and environments versus pleasant and supportive (Yellow) thinking, people, and environments.
