# Supplementary material for: RCT of an integrated CBT-HIV intervention on depressive symptoms and HIV risk
Source: PLoS One. 2017 Dec 14;12(12):e0187180. doi: 10.1371/journal.pone.0187180 (PMC5730221; doi:10.1371/journal.pone.0187180)
Supplement: S2 File — (DOCX) [file pone.0187180.s003.docx]

**RESEARCH PLAN**

#### **PI: Carl A. Latkin Study Title:** The Impact of Neighborhoods, Networks, and Depression on Drug Users’ HIV Risk (NND)

**Sponsor’s Protocol # *1071***

**Research Question:**

This study will implement and evaluate a small group, randomized controlled, phase II intervention to reduce depressive symptoms and HIV risk behaviors among inner city drug users. The intervention will include elements of cognitive behavioral therapy (CBT) for depression among impoverished individuals, emphasizing depressive cognitions and behaviors theorized to be associated with depression and with HIV risk behaviors among mildly to moderately depressed drug users. CBT has been found to be a highly successful, cost-effective approach to depression treatment for low-income minorities, and may be an important approach to reducing HIV risk behaviors among depressed inner city drug users.

This study will also longitudinally examine active drug users’ social and environmental pathways to depression and subsequent HIV risk behaviors. Specifically, we will examine hypothesized mediating/moderating effects of neighborhood characteristics, social network factors, and individual level factors on depressive symptoms and HIV risk behaviors.

This study will include multiple phases: (1) Phase 1: Formative Research to gather information from the target population for materials and curriculum for the intervention, (2) Phase 2: Piloting of project protocols and procedures, and (3) Phase 3: Delivery of the full intervention and data collection. At this time, we are only requesting permission for Phase 1: Formative Research and Phase 2: Piloting.

**We are requesting approval for Phase 3: Intervention delivery and data collection.**

**Rationale:**

Inner-city minority populations have high rates of drug use and HIV/STIs. Although there is ample evidence that African Americans’ health disparities are strongly associated with their social, economic, and neighborhood contexts, few studies have documented pathways through which social and neighborhood contextual factors may facilitate or impede infectious disease risk (Diez Roux et al., 2005; Krueger et al., 1990). This study builds on our prior successful HIV prevention intervention research among inner city, African American drug users, and on promising results of our pilot study of a depression treatment intervention (Hawkins et al., 2005). This study extends our extensive previous findings on the major role of social network characteristics in HIV risk, by adding a detailed examination of the role of neighborhood factors in moderating or mediating depression and HIV risk in this population. This study capitalizes on our prior success in recruiting and retaining this hard-to-reach population in research.

Recent research suggests high rates of depression among inner city African American populations, and that depression mediates effects of social contextual factors and HIV risk behaviors. In cross-sectional studies, we found that neighborhood factors were correlated with depression, which in turn was associated with drug use and HIV risk behaviors (Latkin et al., 2005; Latkin et al., in press, Appendix I). In addition to its relationship to HIV risk behaviors, depression is itself an important public health issue. Depression is the third leading cause of loss of quality-adjusted life years in the U.S. (Unutzer et al., 2000), and is associated with high levels of medical expenditures (Simon, 2003). Depression is also a risk factor for medical non-adherence, coronary heart disease (Rugulies, 2002), type II diabetes, and diabetic complications (Eaton, 2002; Higgins et al, 2006), of which this population is at disproportionate risk.

This study will delineate social environmental pathways that lead to depressive symptoms and HIV risk behaviors. Understanding these dynamics is critical for developing sustainable multilevel interventions for HIV prevention. Another aim of this study is to test a behavioral intervention that targets a key pathway to HIV risk behaviors, namely the path between depressive symptoms and HIV risk behaviors. Although it is often acknowledged that social environmental characteristics are important risk factors, few interventions have assessed how social environmental factors may facilitate or impede behavior change. In this study, we expect that individuals who live in more stressful neighborhoods will be more likely to relapse into depression and sexual and drug-related HIV risk behaviors. We also expect that support network factors, such as emotional support, may ameliorate the negative impact of neighborhood stressors. The proposed intervention will target individuals who have mild to moderate levels of depressive symptoms.

**Aims**

The specific aims of the overall study are:

1. Test the efficacy of a cognitive behavioral treatment (CBT) approach to reduce depressive symptoms and HIV risk behaviors among drug users.
2. Examine neighborhood-, social network-, and individual-level mediators and moderators of change in depressive symptoms and drug and sexual HIV risk behaviors over time among intervention and control condition participants.
3. Examine the relationship between neighborhood factors, network characteristics, and individual-level factors on depressive symptoms, drug use, and HIV risk behaviors among all study participants.

**Methods:**

**Study Design and Rationale**

**Phase 3: Full Trial**

**Phase 3** includes a longitudinal social epidemiological study and a randomized controlled phase II clinical trial. The longitudinal study will include assessments at baseline, 6m, 12m follow-up. A subset of the longitudinal study sample will be eligible for the RCT which will consist of 2 arms- intervention and control condition.

**Population:**

**Phase 3: Full Trial**

Approximately 500 crack smokers and 500 injectors will be recruited to participate in the longitudinal component of the study (i.e. baseline and follow-up assessments) Of these, approximately 250 crack smokers and 250 injectors with CES-D scores ≥16 will be randomly assigned to either the depression intervention or control condition.

**Statistical Analysis Plan**

**Phase 3: Full Trial**

Several data analysis methods will be used to examine the longitudinal data as well as evaluate the intervention by comparing the 2 treatment arms.

For all estimates of the prevalence of behaviors, outcomes (e.g., depressive symptoms) in this population, we will weight the observations to reflect the oversampling of individuals with higher levels of depression for follow-up assessments. For all other models, we will not weight the observations. In testing hypotheses, it is often necessary to adjust for potentially influential covariates that are not the primary focus of the hypothesis. In the case of independent variables that are continuous, we will use multiple linear regression or other modeling strategies to account for covariates. When the dependent variable is dichotomous or polytomous, we will use log-linear or logistic models, or similar techniques. Model fitting will be accomplished using techniques such as hierarchical stepwise regression and the score test to compare nested models. Due to the nature of sampling and study design, there may be some clustering of data, such as clustering by geographic area or intervention group, which can bias estimates. When needed, statistical techniques that allow for analysis of clustered data we will use, such as Generalized Estimating Equations (GEE) to analyze data clustered by neighborhoods (Liang et al., 1996) and Hierarchical Linear Models (HLM) (Byrk et al., 1992) to provide additional information about factors attributable to the level 2 variance. In addition to handling clustering, HLM will be used to conduct analysis with consideration of both individual and contextual factors. Analysis of data at different levels presents methodological challenges, namely correlations of residual errors among observations nested in higher-level units. Such built-in correlation of errors in hierarchical models violates the assumption of independent errors in conventional statistical procedures. However, multilevel or hierarchical analysis takes into account the built-in correlation of errors in multilevel data (Peacher et al., 2004). It has been applied to contraceptive studies (Krull et al., 2001;1999), needle sharing (Korff et al., 1992), and condom use (Morisky et al., 2002).

For several of our hypotheses, often the individual measures of risk behaviors do not meet linearity assumptions. Use of a composite risk score of risk behavior as the outcome measure has been strongly encouraged by Aral and Peterman (1996), and often addresses the problem of non-linearity. Additionally, transformations, such as taking the square root or natural log, can improve the distribution of the risk behavior variables so that their residuals are distributed approximately normally. As the present study is longitudinal in design and different risk behaviors can change in opposite directions, an indicator of the participant’s overall net risk change is most informative. To test separate hypotheses regarding inject and sexual risk behavior, separate composite scores for each will be generated. The composite score for injection risk behavior will include frequency of sharing needles (without cleaning), cookers, rinse water, and cotton. The composite score for sex risk behavior will include frequency of vaginal sex without a condom, anal sex without a condom, exchanging sex for money or drugs, sex with HIV+, sex with IDU, and number of partners in the past 3 months

# Power calculations

# The main outcomes of interest are changes in injection and sexual risk behavior and in level of depressive symptoms. A sample size of 200 in each group is assumed for comparisons of sex risk behaviors and depressive symptoms after two years of follow-up with a 10% attrition rate per year. Only half of the sample will be IDUs, and it is assumed that there will be a sample size of 100 per group with the same attrition rates. There will be approximately 50 cohorts (25 intervention cohorts and 25 control cohorts) of with approximately 8 members with complete data at the end of the two years. The power was calculated for an assessment of intervention effectiveness at the end of the study, when sample sizes will be smallest and thus underestimates likely power for comparisons made earlier in the study. Power calculations were done using NCSS/PASS software. Based on the SHIELD study (IRB# H.30.00.07.17.A), we would expect an average CES-D score of 26.7 and variance of 8.3, given the selection methods proposed in the present study. We estimated power with a 2-sided test, setting the alpha at 0.05, with a total sample size of 400. We expect a small amount of correlation due to intervention group (0.02 in the SHIELD Study). A difference between the two groups post intervention on mean CES-D score of 2.0 provides sufficient statistical power.

|  | **Intercluster correlation** | | |
| --- | --- | --- | --- |
| **Difference in mean CES-D score** | 0.00 | 0.02 | 0.05 |
|  | **Power** | | |
| 2.0 | 0.92 | 0.88 | 0.83 |
| 3.0 | 0.99 | 0.99 | 0.99 |
| 4.0 | 1.00 | 1.00 | 1.00 |

Prior studies suggest a larger effect size for intervention effects on injection as compared to sexual risk behaviors. Consequently, despite the smaller sample size for injection risk comparisons, we expect analyses of the two outcomes to have similar statistical power. We based estimates of expected reduction in risk behaviors on the SHIELD study, creating composite scores for injection risk behavior (needle and cooker sharing and frequency of injecting) and sexual risk behavior (9 risk behaviors). The injection risk score had a mean of 11.6 (SD=4.5). The sex risk composite score had a mean of 1.9 (SD=1.8).

|  | Intercluster correlation | | |  | Intercluster correlation | | |
| --- | --- | --- | --- | --- | --- | --- | --- |
| Diff. in mean injection risk score | 0.00 | 0.02 | 0.05 | Diff. in mean sex risk score | 0.00 | 0.02 | 0.05 |
|  | **Power** | | |  | **Power** | | |
| 1.0 | 0.60 | 0.57 | 0.54 | 0.25 | 0.54 | 0.49 | 0.43 |
| 1.5 | 0.91 | 0.89 | 0.87 | 0.50 | 0.98 | 0.97 | 0.94 |
| 2.0 | 0.99 | 0.99 | 0.98 | 0.75 | 1.00 | 1.00 | 1.00 |
| 2.5 | 1.00 | 1.00 | 1.00 | 1.00 | 1.00 | 1.00 | 1.00 |

**Inclusion and Exclusion Criteria**

**Phase 3: Full Trial**

There are 2 tiers of inclusion and exclusion criteria. The first tier is eligibility criteria for the longitudinal study which will be determined through a brief screening assessment. The second tier is eligibility for the RCT which will be assessed during the baseline survey.

Inclusion criteria for longitudinal study include:

1. 18-55 years old
2. HIV Risk Behavior:

3a. Injected drugs more than 3 times in the past week OR

3b. Smoked crack in the past 6 months AND had 1 of the following sex risks in the past 6 months:

- - - 2 or more sex partners
    - Had a sex partner who injected drugs
    - Had a sex partner who smoked crack
    - Had a sex partner who was HIV+

1. Willingness to attend group sessions

Exclusion criteria for longitudinal study include:

1. Enrolled in another HIV behavioral intervention or depression study in past the 3 years
2. Enrolled in another Lighthouse study in past the 5 years
3. Enrolled in the formative research (Phase 1) or pilot (Phase 2) of the current project.

The 2^nd^ tier will be for randomization into the RCT. These criteria will be assessed during the baseline survey.

Inclusion criteria for the RCT include:

1. Met inclusion criteria for longitudinal study (see above)
2. Have depressive symptoms as determined by CESD scores 16 or greater at baseline assessment

Exclusion Criteria:

1. Severe levels of depression as determined by CESD score ≤40
2. Symptoms of mental illness that require treatment (i.e., actively manic, actively psychotic, DSM IV Axis II Cluster B such as histrionic, borderline, anti-social)

Individuals who are excluded based on mental illness will be given referrals to local agencies that provide mental health services.

In addition, individuals who are disruptive to the group will be removed from the group session and provided with other referrals.

**Gender, Age and Locale**

**Phase 3: Full Trial**

In Baltimore, MD, both males and females will be recruited for the study. All participants will be adults (i.e., 18-55 years).

**Procedures:**

**Recruitment Process**

**Phase 3: Full Trial**

Potential participants will be recruited through outreach, referrals from local agencies, and print advertisements. Trained outreach workers will conduct targeted street outreach in regions of the city that have high levels of drug activity. Recruiters will approach individuals and provide a brief description of the study and give a recruitment flyer and inform interested people to call or visit the Lighthouse to learn more about the study. Posted advertisements displayed at the Lighthouse and other community-based agencies will be used to recruit participants. If needed, we will also place an advertisement in local newspapers. (see attached recruitment flyer)

**Study Procedures in Sequential Order**

**Phase 3: Full Trial**

***Screening***

Individuals who are interested in the study will be screened over the telephone or face-to-face by a trained interviewer in a private office. They will provide verbal consent prior to being administered a brief screening assessment to determine eligibility into the study (see attached screener).

***Baseline interview***

Eligible participants will be sent a Baseline appt reminder letter (see attached letter). Partiicpants who miss their baseline visit will be sent a Missed appointment letter (see attached letter).

Participants will be asked to complete a baseline visit in a private office at the Lighthouse. The baseline visit may take up to 2 ½ hours to complete. After the participant has provided written consent, the baseline visit will begin and consist of the following components:

1) Collection of Locating information: Participants will be asked for information that will be used to try to remain in contact with them to remind about program sessions and follow-up visits. Participants will be asked to provide their names, date of birth, addresses, and telephone numbers. In addition, participants will be asked to provide contact information of at least one person if they are unable to provide their own contact information (e.g., homeless, transient populations). We will try to get as much other locator information as possible for each participant. However, we will not press for additional information if the participant is uncomfortable or distrusting of our motives. Participants will be asked if they are interested in being contacted after the end of the study about future studies. Information will be entered into a Client Locating and Scheduling Database which is a separate database from the survey data. Electronic contact information for participants who do not wish to be contacted in the future will be destroyed 1 year after the study ends by the Data Manager.

2) Survey Administration: A survey will be administered in part by a trained interviewer and a portion will be administered using Audio Computer Administered Self Interview (ACASI) software. Prior to the start of the ACASI portion, the interviewer will show participants how to use the computer and offer assistance if needed.

The survey will include questions on several domains including HIV risk behaviors, physical and mental health history, neighborhood characteristics and drug history. The survey will also include a social network inventory. A network inventory is a commonly used instrument that is used to measure social support and social integration, and diffusion of health information. A modified version of Barrera’s support network questionnaire will be utilized (Barrera, et al., 1981). Participants will be asked to use first names and last initials, nicknames, or initials of those they name in order to keep clear who they are talking about during the questionnaire yet minimize identifiable information. See attached survey. The survey items will also assess eligibility into the RCT. Participants will also be notified of their eligibility for randomization (based on responses to survey items) during the baseline survey.

The survey administration component of the visit will be audio-recorded so that we can conduct quality assurance of the data and ensure that interviewers are following the standard protocols. The audio file will be saved in mp3 format and saved to a password-protected computer. The audio file will be labeled with the study ID of the participant and the type of survey (e.g. 3001baseline.mp3)

4) Testing for Opiate and Cocaine Metabolite: Participants will be asked to provide an oral specimen to test for cocaine and opiate metabolites using the Intercept Oral Collection Device. The purpose of testing for these drugs is to validate drug use measures from multiple sources such as self-reported disclosure. The specimen will be collected using a cotton swab that the participant places in their mouth for 3-5 minutes. The participant’s name will not appear on the specimen or any transmittal forms. Participants will also be informed that refusal to take the test will not affect their participation in the study. Results of this test will not be provided to the participants. The specimen will not be stored and will be destroyed after analysis.

5) Testing for HIV: Participants will also be asked to provide an oral specimen to test for HIV. The purpose of conducting the HIV antibody test is to measure HIV status. Participants from prior studies have also identified testing as a valuable service and benefit to their participation. Participants will be tested for HIV antibody using the OraQuick Advance oral specimen collection device. State and Federal licensure has been obtained to conduct “rapid” HIV testing at the Lighthouse.

Study staff who will be collecting the specimens and providing prevention counseling have received training by the OraSure Technologies Company and the Maryland State AIDS Administration. Following completion of the survey, the participant will receive counseling and complete the OraQuick Advance test. Participants will have the OraQuick oral fluid rapid HIV test administered consistent with instructions included in the test packaging. This is a CLIA-waived test and includes 2 steps before reading a result . For each participant, an oral fluid specimen will be obtained from the mouth using the OraSure oral specimen collection device by swabbing the upper and lower outer gums. The device will then be inserted and stirred into the OraQuick developer solution and vial. Next, the OraQuick test indicator will be inserted into the vial. Typically, test results will be available within 20-40 minutes. During this period, participants will receive prevention counseling in accordance with study protocols. After the result is read, the specimen will not be stored and will be properly disposed at the clinic.

Participants whose result is negative will be notified of their negative test result and will receive prevention counseling. Participants whose test result is reactive will be informed of their preliminary positive test result and will receive prevention counseling. Participants who are learning for the first that time that they may be HIV positive (preliminary positive) will be asked to consent to have 6 cc’s (1 teaspoon) of blood drawn from their arm by a trained phlebotomist to be sent to a lab at the Maryland Department of Health and Mental Hygiene for confirmatory testing. After testing, the blood specimen will not be stored and will be destroyed by the testing lab. The result of a Western Blot test performed on this sample will serve as confirmation of their HIV status and are available in approximately 7 days. Participants will be scheduled to return for the result of this test approximately 7 days after the enrollment visit (depending on local lab schedule). If this confirmatory test is positive participants will be referred to appropriate counseling and medical services.

All participants both those who test HIV positive and negative are eligible to take part in the study. The Lighthouse has a comprehensive resource guide that includes agencies that provide services ranging from healthcare, drug treatment, and housing. Some agencies listed in the compendium are the JHU Moore Clinic, Baltimore City Health Department, and Healthcare for the Homeless.

Through permission of the Maryland State AIDS Administration researchers are provided with exemption from reporting HIV test results pursuant to COMAR 10.18.02.02. One requirement for the exemption is local IRB approval of the project. After receiving JHSPH IRB approval, an application for exemption will be submitted. Exemptions for other studies conducted at the Lighthouse have been received (most recently Unity in Diversity, IRB# 559). Despite this exemption, the number of individuals tested for HIV and the number tested positive are required to be provided to the AIDS Administration These reporting dates are dictated by the AIDS Administration.

## Partner Notification Services

In addition to testing, a limited contact tracing *service* will be provided (referred to as Partner Notification) to our HIV positive participants. Partner notification is a public health service aimed to reduce the spread of HIV and to ensure that HIV seropositive individuals receive timely medical care. Partner notification is offered as a service and is separate from the research activities. The goal of this service is to increase the number of people who may have been exposed to HIV to get tested but is not related to any of the research aims.

Early testing, diagnosis, and treatment for HIV can dramatically reduce the morbidity associated with HIV. Partner notification is a standard method for identifying persons at risk of infection. The term “partners” refers to sex and injection partners. In all cases of partner notification, the identity and confidentiality of the participant is protected. Cards from the Baltimore City Health Department will be used to notify partners that “in the interest of their health, they are urged to attend one of the local Baltimore City health clinics to be tested”.

If a participant reports being HIV positive or tests HIV positive as part of the study, they will be informed about the importance of Partner Notification as a public health approach to stop the spread of HIV and to increase the numbers of people who know their status. They will be offered the following three options, which are strictly voluntary. If participants are concerned about consequences of Partner Notification such as potential violence they will be referred to the Baltimore City Health Department for their assistance in notifying partners.

*Partner Notification Options*

- *Participant initiated*: Participant will be provided with Partner Notification cards, which are based on those used by the Baltimore City Health Department. Participants can deliver these cards to their drug and sex partners who may be at risk for acquiring and transmitting HIV. These cards do not identify the participant as the potential source of HIV.
- *Clinic mailing*: Envelopes and postage will be provided which will enable a participant to mail out these cards to their partners. We would also inform the participant that their name will not included on the notification card.
- Participant can *decline* the above mentioned options. In such cases, participants will be provided with information about services that the Baltimore City Health Department offers for HIV testing and medical care. Participant will also be encouraged to have their partners tested for HIV.

***Randomization***

After approximately 20 individuals have been deemed eligible for the intervention, an intervention group will be scheduled. Eligible participants will be contacted by phone and mail to remind them about the upcoming group session. Attached is the group reminder letter.

On the first day of the intervention, participants who show up will be randomized into the intervention or control group. The Data Manager will assign group membership to each index using a computerized randomization program.

***Intervention***

This study will include 1 treatment and 1 control condition. The treatment group is a 10-session skills-building workshop (9 group-based sessions and 1 individual session) based on Cognitive Behavior Therapy. The intervention will focus on developing skills to cope with stress and lower one’s risk for HIV. The intervention group will be led by 2 trained group leaders.

The control group will be a 1 session on standardized HIV information. This session will be led by 1 group leader and last approximately 90 minutes.

***Follow-up Visits***

Follow-up surveys will be conducted at 6 and 12 months after the intervention. All participants who meet the longitudinal study eligibility criteria will be invited to participate in the follow-up visits. Participants will be informed of these follow-up visits during the informed consent process at the baseline visit. Approximately 1 month before their follow-up date, staff will contact clients through phone calls and letters to remind them of upcoming follow-up assessments. A copy of the reminder letter for each follow-up period is included.

**Methods of Intervention**

*Methods for Dealing with Adverse Events*

Study staff will be trained to identify and manage adverse events (e.g., participants becoming mentally distressed, carrying weapons, using drugs and alcohol, or becoming ill). Study staff have attended trainings given by the Maryland AIDS Administration, Baltimore Crisis Response (BCR), and the House of Ruth, a local domestic violence program.

The Lighthouse has established collaborative relationships with a variety of local resources such as local drug treatment, mental health, and other service providers who have facilitated referrals for past Lighthouse study participants. We also maintain an extensive community resource database that is updated several times throughout the year. We work closely with the Johns Hopkins Hospital and the Baltimore Crisis Response (BCRI), a community-based mental health service provider. Individuals who report very high levels of depressive symptoms will be referred to the BCRI. If participants report suicidal ideation or otherwise indicate psychiatric crisis, the interviewer will notify Dr Tobin (Co-Investigator) or Dr. Davey-Rothwell (Project Director) who will facilitate his/her call to the BCRI for evaluation. Upon participant request, BCRI will send a counselor to evaluate the participant and, if indicated and requested, bring him/her to their facility for emergency residential psychiatric treatment. Drs Tobin and Davey-Rothwell will refer questions on the study’s psychiatric emergency cases or protocol to the project’s psychiatrist, Dr. Larry Wissow (Co-PI), who will be on call for psychiatric issues and emergencies. Dr. Wissow, has developed the mental health crisis protocols for the Lighthouse. All project facilitators and interviewers have received mental health crisis training.

Participants who experience significant distress will be referred to a community-based crisis center, Baltimore Crisis Response (BCR) which is an agency with whom the study investigators have an ongoing relationship. BCR have beds available for referral and trained staff who take phone calls. If unexpected events occur during a focus group or pilot intervention session, group leaders will be trained to work as a team so that as one addresses the immediate concern, the other will continue to lead the group. Group leaders will not be expected to be mental health professionals. Instead, they will be able to identify situations where it is necessary to get help from other trained staff. In mental health emergencies, participants will be referred to clinical staff on site or to clinical care in the community and known to the research team.

**Methods for Dealing with Illegal Reportable Activities**

Participants will be informed that Maryland law requires that cases of child abuse (physical or sexual), reports of harming self or others will be reported to Baltimore City Social Services and/or the Baltimore City Police. The survey does not contain questions about abuse, so it is unlikely that this topic will be broached during the interviews or focus groups. Upon approval of Phase 3 of this study by the JHSPH IRB, we will seek a Certificate of Confidentiality from the National Institute on Drug Abuse.

**Risk/Benefits:**

**Description of Risks**

The researchers will keep the information gathered from participants as confidential as possible, but complete privacy cannot be absolutely guaranteed. There is the potential risk that information shared by a participant during a focus group or pilot group session would be shared outside of the group by another participant. The consent form indicates that information about other participants should not be shared outside of the group, but we cannot guarantee that other intervention participants will keep information that is shared in the groups confidential. Some participants may feel uncomfortable talking in a group setting (i.e. focus groups or pilot sessions) with strangers. In addition, some people may feel guilty, anxious, upset, or depressed because of the information shared by themselves or others.

Enrollment in a group for behavioral intervention involves minimal risk to participants. The voluntary informed consent form and process describe all of the potential risks of study participation. Some participants may feel uncomfortable talking in a group setting with strangers. Participants experiencing significant distress or requesting services will be assisted immediately and referred to the appropriate supportive services. In a health or mental health emergency situation, participants will be referred to clinic staff on site (a psychiatrist on faculty who consults with the project) or to emergency care in the immediate community (e.g. Baltimore Crisis Response). Either the consulting psychiatrist will come to the study site to meet with and assess the client or a trained staff from Baltimore Crisis Response will speak with the client on the phone to assess and make referrals or can send the Baltimore Crisis Response Mobile Team to assess in person. During the consenting process, participants will be informed that 1) cases of child abuse and 2) imminent harm to self or others will be reported to the Baltimore City Police and the Baltimore Department of Social Services as appropriate. The intervention sessions do not contain questions about child abuse, so it is unlikely that this topic will be broached.

Some participants may feel uncomfortable talking in a group setting with strangers. In addition, some people may feel guilty, anxious, upset, or depressed because of the information shared by themselves or others. Some participants who are randomized to the control condition may feel disappointed that they are not able to partake in the full intervention.

**Description of Measures to Minimize Risks**

To minimize risk of breaches of confidentiality, we have taken a number of steps, including: 1) making clear all of the potential risks of study participation during the informed consent procedure and letting participants know during the informed consent procedure that they have the freedom to withdraw from the study at any time; 2) intending to request a Certificate of Confidentiality that will protect the identities of participants in this study forever, even from legal subpoena; 3) not using individual identities in any reports or publications that may result from this research; 4) storing all audio recordings of interviews, focus groups, pilot visits, baseline and follow-up visits, and intervention sessions and quantitative data on a password protected server with firewall; and 5) using unique study numbers on all data collection instruments.

**Description of Potential Benefits**

Few direct benefits to participants are anticipated during Phase 1 and Phase 2. Prior experience suggests, however, that participation in focus groups and pilot intervention sessions is an enjoyable experience for some participants and that many feel good about themselves as a result of helping researchers address issues related to HIV prevention. Referrals to mental health, counseling and other supportive services will be provided, along with printed information about the prevention of HIV transmission to all participants. Finally, some participants may learn tools to help cope with stress or may learn how to change risky behaviors

In Phase 3, the potential benefits will be learning about HIV and mental health. Referrals to mental health counseling, drug treatment, and social services will be available to clients who are in need of these services. Participants who take part in the intervention/control sessions may find the sessions informative and enjoyable. In addition, some participants may learn tools to help cope with stress or may learn how to change risky behaviors

**Description of Level of Research Burden**

Interviews, focus groups, pilot visits, baseline and follow-up interviews, and intervention sessions will be scheduled at times convenient to the participants. The focus groups, interviews, and pilot visits should not take more than 2.0 hours to complete. The baseline and follow-up visits may take up to 2 ½ hours to complete and the intervention sessions will take approximately 90 minutes. Participants are free to withdraw from the study at any time and will be informed of this right during the informed consent statement.

**Compensation:**

**Type of Compensation**

**Phase 3: Full Trial**

Participants will be paid with checks that can be cashed at local check cashing stores.

**Amount of Compensation**

**Phase 3: Full Trial**

Participants will be paid $35 for completion of the baseline and the follow-up visits. In addition, they will be paid $25 for attending each group session.

**Schedule of Compensation**

**Phase 3: Full Trial**

Participants will be paid immediately after completing a baseline, follow-up or intervention/control group session.

**Disclosure/Consent Process:**

**Description of the Consent Process**

In Phase 3, participants will meet individually with a staff member in a private room to go through the informed consent process. The interviewer will read through the consent form with the client.

A participant will be asked if they are willing to provide an oral specimen to test for cocaine and heroin metabolites. They will indicate their willingness by completing a box on the consent form. In addition, they will be asked if they are willing to provide an oral sample to test for HIV. They will indicate their willingness by completing a box on the consent form.

Participants who are interested in participating in the study will also indicate their willingness by signing the consent form.

Before a participant signs the consent form, staff will thoroughly review the form, ask if the participant understands the content of the consent form, allow the participant time to read the consent themselves and answer any questions they may have. Participants will be given a copy of the informed consent form for their records.

**Who Will Obtain Consent**

Interviewers and Group Leaders will obtain consent. All staff have completed the requirement IRB training modules.

**Where and When**

Written consent will be obtained at the beginning of the baseline visit. Consent will be obtained in a private room at the Lighthouse research facility.

**Safety Monitoring:**

A DSMB has been established for this project.

***Membership***

The DSMB has 4 members with a range of expertise:

1. Dr. Christopher Welsh- board certified psychiatrist and Associate Professor in the School of Medicine, Department of Psychiatry. He is also the Director of a drug treatment clinic, which serves predominately heroin users in Baltimore.
2. Dr. Bernadette Cullen, MB, BCh, BAO, MRCPsych Assistant Professor Johns Hopkins School of Medicine. Dr. Cullen is the Director of the Community Psychiatry Program at the School of Medicine.
3. Dr. Bruce Herman, PhD. is a clinical psychologist and Assistant Director of Outreach & Consultation at Towson University.
4. Dr. Elizabeth Stuart, PhD. Assistant Professor in the Department of Mental Health, Johns Hopkins Bloomberg School of Public Health. Dr. Stuart is trained as a biostatistician and her research interests are examining the development and use of methodology to better design and analyze the effects of public health and educational

***DMSB Charge or Charter***

The primary responsibility of the project’s DSMB is to safeguard the interest of clients. Therefore, the DSMB have been charged with the following actions:

- Provide review of the protocol and the data safety monitoring plan (DSMP).
- Be available to the Investigator for consultation concerning any adverse study events.
- Review the progress of the study carefully and diligently with special attention to reports of adverse events, drop-outs, or perhaps some other reports generated by group leaders
- Provide a written report to the IRB which summarizes oversight activities and recommendations, and any concerns regarding subject safety.
- Consider the impact of newly published findings bearing on the safety profile of the study.

***Plans for providing DSMB reports to the IRB:*** A brief DSMB report including a summary of their activities will be submitted to the JHSPH IRB when the project annual report is submitted.

***Describe plans for interim analysis and stopping rules.***

Interim analyses of the data will be conducted halfway through the follow-up period (and where virtually all participants will have provided 6-month behavioral interim behavioral endpoints). Interim reports will be prepared for the DSMB by an external statistician; they will be presented to the protocol team blinded as to study arm (Arms will be labeled simply “A”, and “B”,).

If the results show any of the following:

- Increases in the overall number of drug use and risk behaviors for all study participants, as compared to baseline levels, that are statistically significant at the p<.001 level.
- Differences in study outcomes between the control arm and intervention study arm that are statistically significant at the p<.001 level
- Differences in the number of serious adverse events (calculated by collapsing all SAEs based on statistical power assessments) between the control arm and intervention study arm that is statistically significant at the p<.01 level. SAEs include mortality, suicide, suicide attempts, hospitalizations (physical health and mental health or drug-related), and violence.
- Level of depression symptoms (CES-D_) will also be assessed between the two arms to ensure that the experimental intervention does not lead to greater symptomology

Then consideration will be given to stopping the study. When considering whether to stop the study, the overall risks and benefits experienced as of the interim analysis date will be weighed by the protocol team. The recommendation of the DSMB and opinion of the study sponsor will be given heavy weight. In addition, we will track important events and consider appropriate modifications in procedures. We will consult with the DSMB regarding any substantive changes in study procedures.

**Confidentiality Assurances:**

***Certificate of Confidentiality (if applicable)***

Upon approval from the JHSPH IRB of Phase 3 of the study, we will seek a Certificate of Confidentiality from the National Institute on Drub Abuse.

***Data Security***

All participants who consent to participate in the Phase 3 will be assigned a unique identifier. Their survey data, locating information, which will be collected electronically, and information regarding sessions that they attend will be entered into a password protected database that is located on a secure internal server. Study staff, Investigators, Data Manager and Data Analysts will have access to the database. We will use this information to track the participants for reminder calls for follow-up visits and to exclude participants from future screening and duplicate enrollment in the full trial phase. The locating data files will be destroyed by the Data Manager 6 months after the study is over.

Digital audio-recorded interviews, focus groups, pilot visits, baseline, follow-up surveys, and intervention sessions will be verbally labeled with the date of visit and participant’s unique study ID number. These data files will be saved on a password protected computer with firewall server and labeled with the date and number of the focus group. These recordings will be deleted by the Data Manager within 1 year after the study ends. During the focus groups, pilot group sessions, and full trial group sessions, participants will be asked to use only their first names when identifying themselves. Attendance lists will collect only the first names and last initial of participants as a way to monitor attendance and attendance information will be entered into the participant database immediately after the intervention and the paper form will be destroyed.

***Plans for Record Keeping***

**Person Responsible & Telephone Number**

Dr. Carl Latkin is the Principal Investigator for the study (955-3972). Joanne Jenkins is the Data Manager (502-5368).

**Where Data Will Be Stored for Data Security**

All data will be stored on an internal server that is password protected with a firewall. All behavioral data will be identified with only a unique study ID number and no names will be used.

**Who Will Have Access to the Data**

Study Investigators: Carl Latkin, Karin Tobin, Larry Wissow, and Amy Knowlton; student investigators: Danielle German, S, Janet Kuramoto, Samantha Illangasekare, and Jennifer Pearson and the Project Director: Melissa Davey-Rothwell; and Data Manager: Joanne Jenkins will have access to the data. All have completed the CITI training on conducting research with Human Subjects.

**Plans for Disposition of Identifiers at End of Study**

Locator information will be deleted by the Data Manager 6 months after the study ends. The audio-recordings of the in-depth interviews, focus groups, pilot sessions, baseline and follow-up sessions, and intervention sessions, will be destroyed 1 year after the study is over.

**Plans for Destruction of the Data/Samples at the End of the Study**

The Data Manager will delete all recordings of the focus groups, interviews, pilot sessions, baseline and follow-up visits, and intervention sessions 1 year after the study is over. The deleted files will then be purged from the computer recycle bin.

**Collaborative Agreements:**

Not applicable

**Other IRB Approvals:**

Not applicable
